# MyKidneyCoach, Patient Activation, and Clinical Outcomes in Diverse Kidney Transplant Recipients: A Randomized Control Pilot Trial

**DOI:** 10.1097/TXD.0000000000001462

**Published:** 2023-03-15

**Authors:** McLean D. Pollock, Nicolas Stauffer, Hui-Jie Lee, Shein-Chung Chow, Ito Satoru, Lynnette Moats, Sherri Swan-Nesbit, Yan Li, John K. Roberts, Matthew J. Ellis, Clarissa J. Diamantidis, Sharron L. Docherty, Eileen T. Chambers

**Affiliations:** 1 Department of Psychiatry, Duke University, Durham, NC.; 2 Department of Biostatistics and Bioinformatics, Duke University, Durham, NC.; 3 Department of Surgery, Duke University, Durham, NC.; 4 Department of Pathology, Duke University, Durham, NC.; 5 Department of Medicine, Duke University, Durham, NC.; 6 Department of Pediatrics, School of Nursing, Duke University, Durham, NC.; 7 Department of Pediatrics, Duke University, Durham, NC.

## Abstract

Kidney transplant (KT) recipients who are not actively engaged in their care and lack self-management skills have poor transplant outcomes, which are disproportionately observed among Black KT recipients. This pilot study aimed to determine whether the MyKidneyCoach app, an mHealth intervention that provides self-management monitoring and coaching, improved patient activation, engagement, and nutritional behaviors in a diverse KT population.

**Methods:**

This was a randomized, age-stratified, parallel-group, attention-control, pilot study in post-KT patients. Participants were randomized into the attention-control with access to MyKidneyCoach for education and self-management (n = 9) or the intervention with additional tailored nurse coaching (n = 7). Feasibility, acceptability, and clinical outcomes were assessed.

**Results:**

The acceptability of MyKidneyCoach by System Usability Scale was 67.5 (95% confidence interval [CI], 59.1-75.9). Completion rates based on actively using MyKidneyCoach were 81% (95% CI, 57%-93%) and study retention rate of 73%. Patient activation measure significantly increased overall by a mean of 11 points (95% CI, 3.2-18.8). Additionally, Black patients (n = 7) had higher nutrition self-efficacy scores of 80.5 (95% CI, 74.4-86.7) compared with 75.6 (95% CI, 71.1-80.1) in non-Black patients (n = 9) but lower patient activation measure scores of 69.3 (95% CI, 56.3-82.3) compared with 71.8 (95% CI, 62.5-81) in non-Black patients after 3 mo.

**Conclusions:**

MyKidneyCoach was easy to use and readily accepted with low attrition, and improvements were demonstrated in patient-reported outcomes. Both Black and non-Black participants using MyKidneyCoach showed improvement in self-management competencies; thus, this intervention may help reduce healthcare inequities in KT.

Compared with dialysis, kidney transplantation (KT) offers superior quality of life and decreased morbidity for patients with end-stage kidney disease.^1,2^ Yet despite advances in transplant care and immunosuppression, KT clinical outcomes remain suboptimal and are disproportionately observed among Black transplant recipients who, compared with White recipients, suffer more allograft rejection and dysfunction.^3-13^ Although reasons for these differences are multifactorial, issues related to inadequate patient activation and self-management skills play a significant role.^14,15^ One-third of KT failures and 125 000 annual deaths can be attributed to inadequate patient activation and self-management skills, with an incremental lifetime cost of $78 709 per patient and loss of 1.66 quality-adjusted life years.^16,17^

Posttransplant management consists of multidrug regimens and consistent healthcare navigation, including adhering to medications, monitoring for signs of rejection, maintaining a healthy lifestyle, and sustaining emotional health and stability.^15,18^ Thus, KT recipients must develop self-management skills and become active and engaged patients to manage their health effectively and remain vigilant over potential complications of transplantation, including the risk of graft loss and future comorbid conditions.^15,18^ There is a critical need for interventions that improve patient activation and self-management skills after transplant to mitigate inequities in allograft loss.

Coaching and mobile health (mHealth) technologies for self-management and medication adherence have been shown to reduce hypertension, improve glycemic control,^19,20^ and improve cancer pain management, especially in Black patients with these chronic diseases.^21,22^ Notably, Black patients with chronic kidney disease, the same pool of patients who require KT, are more receptive to mHealth than their nonminority counterparts and think that mHealth can help them become engaged in their care.^23,24^ Therefore, we hypothesized that the incorporation of both mHealth and coaching has the potential to increase the sustainability of either intervention alone, lead to increased patient activation and self-management skills, and improve inequities in transplantation. In a pilot study, we aimed to determine the feasibility and acceptability and identify facilitators and barriers of MyKidneyCoach, a self-management application (app) alone or paired with clinical nurse coaching, in a predominantly Black and White KT population. We also aimed to determine the impact of MyKidneyCoach on patient-reported outcomes (patient activation, self-management, and nutrition self-efficacy) and clinical outcomes (allograft function, de novo donor-specific antibody [DSA] development, acute rejection, mortality, graft survival, and hypertension) and whether the effects of our intervention varied by race.

## MATERIALS AND METHODS

### Patients and Study Design

**FIGURE 1.**
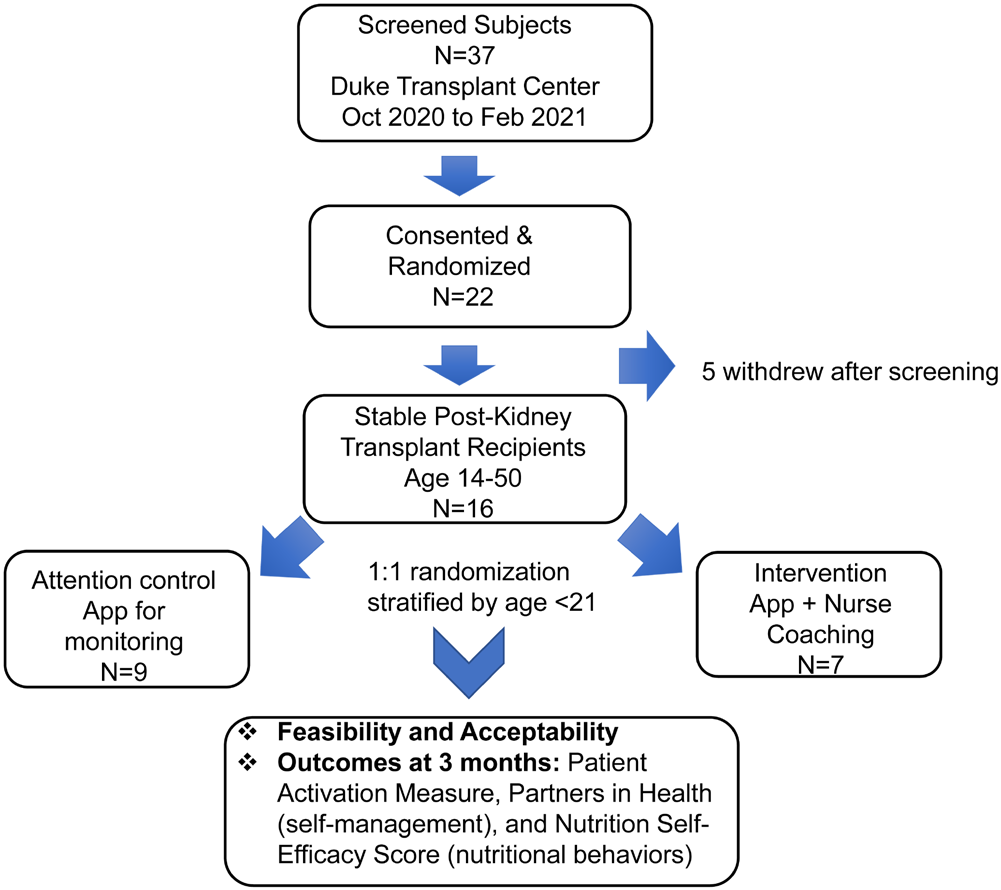
Study flowchart. Represented is the flowchart and design of the pilot study.

This was a single-institution, age-stratified, randomized, parallel-group, attention-control pilot study in patients undergoing their first KT, age 14 to 50 y, followed at Duke University Medical Center (ClinicalTrials.gov NCT04382248). Patients were recruited from October 2020 to February 2021 and were followed until September 2022. Because of coronavirus disease 2019 (COVID-19) pandemic, patient recruitment was limited to telephone and/or e-mail contact. Target enrollment for this trial was 40 subjects (20 subjects per arm). Inclusion criteria consisted of stable allograft function (serum creatinine increase of no >25%) defined as follows: transplanted for a minimum of 1 mo but not >3.5 y; tacrolimus and/or sirolimus based immunosuppression; smartphone ownership; the absence of preformed DSA; and patient activation measure (PAM) level between 1 (disengaged and overwhelmed) and 3 (taking action), to include only those KT recipients with suboptimal patient activation. Patients were excluded if they had a rejection episode or DSA in the blood before or at the time of enrollment and had development delay or psychological impairment that prevented them from using a mobile app. Study flowchart and design are depicted in Figure 1. Eligible subjects who met inclusion/exclusion criteria were randomized 1:1 into one of the 2 following study arms, stratified by adolescents (age <21 y) versus adults (age ≥21 y): the attention-control arm, which is used to disentangle the benefits of the attention gained from being in a study from the impact of the intervention itself,^25^ had access to MyKidneyCoach for education and self-management, whereas the intervention arm actively used MyKidneyCoach for education, self-management, and tailored text and telephonic clinical nurse coaching. This stratified randomization scheme optimized the balance among treatments for age, which was considered to be associated with mobile app usage. Study visits were at the time of enrollment (baseline) and 3 mo. The protocol Pro00104469 complies with the Declaration of Helsinki and the Declaration of Istanbul. All participants signed a written informed consent approved by Duke University Institutional Review Board.

### MyKidneyCoach

The MyKidneyCoach intervention comprised (1) a self-management app that provided educational materials and monitoring of posttransplant care using a smartphone and (2) personalized, text-based, and telephonic coaching from a trained clinical nurse triggered through the app to promote patient activation and self-management after KT. Study staff helped participants download the Apple or Android-compatible self-management app through the Duke Electronic Health Record, MaestroCare. To develop MyKidneyCoach, we used Pattern Health, a mHealth platform, which was compliant with the Health Insurance Portability and Accountability Act and the Health Information Trust Alliance Common Security Framework. The participant was oriented to the app and shown how to access educational videos^26^ and newsletters, which covered both transplant-specific and general topics of monitoring/understanding symptoms, laboratory and transplant complications, scheduling/attending appointments, medication understanding/adherence, communication with provider, nutrition, hydration, exercise, relaxation, and emotional health. Additionally, the app provided monitoring tools to track pain, medication taking, blood pressure (BP), exercise, sleep, and food/water intake. Finally, study participants completed surveys to assess patient activation, self-management, and nutrition efficacy. These data were transmitted to a secure Pattern Health Server from the app and subsequently downloaded to the secure RedCap database.

The coaching intervention, based on Deci and Ryan’s Self-Determination Theory,^27^ was delivered individually by the same clinical research nurse, who was trained for 6 wk using a tested curriculum, including active listening skills, motivational interviewing techniques, and the health coaching process throughout the study. This was developed by McLean Pollock, PhD, a certified Health and Wellness Coach. The coach and participant communicated by 30-min phone call every other week for 3 mo and text messages via the app scheduled between the phone calls. This allowed participants flexibility and convenience to be in a location of their choice. Initially, the coach elicited the participant’s long-term goals and related values of optimal health and wellness within the posttransplant context as a guidepost for coaching and the development of the participant’s motivation for change. As the participant identified a vision of health and wellness, the coach elicited the participant’s sense of self-efficacy and competence in making a behavior change through the development of incrementally, progressive steps, which empowered the participant to take leadership in managing their health. The curriculum allowed the coach to help the participant identify goals and tailor coaching for change within the domains of posttransplant self-management, as previously described. Intervention fidelity was assessed by tracking the number of times patients used the app, defined as entering into the app, and participating in coaching sessions, as well as the length of each session. This was performed by the clinical research nurse and confirmed by McLean Pollock, the intervention supervisor.

### Feasibility and Acceptability

Feasibility was measured through (1) whether the target sample size was achieved, (2) recruitment rate, and (3) completion rate at 3 mo. The recruitment rate per month was defined as the number of participants recruited divided by the total number of months of recruitment. The completion rates were defined as the number of participants who completed at least 1 question in the System Usability Scale (SUS) survey, PAM survey, or were actively using the app at 3 mo, divided by the number of participants recruited. Acceptability of the overall functionality of the intervention was measured through the validated 10-item SUS (score range, 0–100) at 3 mo, which has been used extensively in KT mHealth studies.^28-30^

### Patient-reported Outcomes

The primary patient-reported outcome was a change in patient activation as measured by PAM^31,32^ from baseline to 3 mo. PAM is a 13-item scale (score range, 0–100) of patient activation in healthcare that has been used in KT trials with reliability, validity, test–retest stability, and accuracy.^33,34^ Secondary patient-reported outcomes included change from baseline to 3 mo of self-management by Partners in Health (PIH, 12-item scale; score range, 0–96),^35,36^ and nutritional behaviors measured by Nutrition Self-Efficacy Scale (20-item measure; score range, 0–100).^37,38^

### Posttransplant Immunosuppression, Monitoring, and Clinical Outcomes

All participants were on maintenance steroid-free or steroid-based immunosuppression with either tacrolimus and mycophenolate mofetil or mycophenolic acid, or belatacept and sirolimus. Serial monitoring of KT function (serum creatinine), DSA (Luminex assay), biopsy-proven rejection graded by 2019 Banff criteria,^39^ immunosuppressant levels (tacrolimus or sirolimus), BP (hypertension defined as BP >130/80 mm Hg based upon American Heart Association Guidelines^40^), body mass index, graft survival (defined as not returning to dialysis), and patient survival were followed for the 3-mo study period.

### Performance of Semistructured Interviews

We completed the qualitative phase of the MyKidneyCoach, which comprised 3 semistructured telephone interviews with racially diverse KT recipients (White, Latinx-X, Black), who completed the app+coaching intervention arm, to evaluate the intervention. Open-ended questions, including asking about their general experience with coaching, the coaching relationship, and use of the app, barriers to using the app or suggested areas of improvement related to both the app and coaching model, as well as the perceived impact of receiving app-based coaching from a nurse, were asked to prompt discussion regarding MyKidneyCoach intervention experience. The study team used qualitative content analysis^41,42^ to classify qualitative data into similar and disparate concepts to describe the facilitators and barriers to the use of the MyKidneyCoach app and intervention.

### Statistical Analysis

For sample size justification, we assumed a mean of 44 in the PAM score with a standard deviation of 6 for both groups before intervention based on preliminary data. The correlation between baseline and postintervention scores was assumed to be 0.8. After adjusting for baseline scores in an analysis of covariance analysis (ANCOVA), a total sample size of 28 participants (14 in each group) achieves >80% power to detect a 3-point increase based on preliminary data (from 44 to 47) in the intervention group compared with control group using an F test at the 5% level of significance. Twenty patients in each group (a total of 40) were planned to account for potential study drop-out.

This analysis was performed on the basis of the intention-to-treat population, which was defined by all randomized subjects who had at least 1 follow-up evaluation. Descriptive statistics were used to summarize outcomes and demographic/clinical characteristics. The recruitment rate per month and the 95% confidence interval (CI) were estimated using a Poisson distribution. The completion rates were estimated with binomial distribution and Wilson 95% CIs. The mean SUS score and the 95% CI of the mean were constructed from a t-distribution for all participants and also stratified by intervention. One-way ANCOVA analyses adjusting for the baseline scores were conducted to compare PAM, PIH, and nutrition self-efficacy scores at 3 mo between intervention and control groups and between Black and other races using the mean group difference and its 95% CI. Statistical significance was assessed at the 0.05 level without adjusting for multiple testing. All statistical analyses were conducted using R version 4.0.0 (R Core Team, Vienna, Austria).

## RESULTS

### Demographics of the Pilot Study

Patient demographics and clinical characteristics are provided in Table 1. Overall, the control and intervention groups had similar distributions of sex, race, ethnicity, and donor type. Additionally, immunologic characteristics such as calculated panel reactive antibody, HLA mismatch between donor and recipient, and immunosuppression were similar (Table 2). However, the intervention group was older, with a mean age of 41.6 y, and was more often treated with belatacept, a monthly intravenous infusion, compared with the control group.

**TABLE 1.** Patient demographics

	**Control (N** = **9**)	**Intervention (N** = **7**)	**Overall (N** = **16**)
Age ≥21 y, n (%)	8 (89)	6 (86)	14 (88)
Age (y), mean (SD)	35.9 (12.4)	41.6 (11.6)	38.4 (12)
Female, n (%)	3 (33)	5 (71)	8 (50)
Race			
White, n (%)	4 (44)	3 (43)	7 (44)
Black, n (%)	4 (44)	3 (43)	7 (44)
American Indian/Alaska Native, n (%)	1 (11)	0 (0)	1 (6)
Not reported/declined, n (%)	0 (0)	1 (14)	1 (6)
Ethnicity			
Non-Hispanic, n (%)	9 (100)	6 (86)	15 (94)
Hispanic, n (%)	0 (0)	1 (14)	1 (6)
Donor type			
Living donor, n (%)	4 (44)	3 (43)	7 (44)
Donor after brain death, n (%)	3 (33)	3 (43)	6 (38)
Donor after circulatory death, n (%)	1 (11)	1 (14)	2 (13)
Missing, n (%)	1 (11)	0 (0)	1 (6)
Years since transplant, mean (SD)	1.7 (1.0)	2.2 (1.1)	1.9 (1.0)

SD, standard deviation.

**TABLE 2.** Immunologic characteristics

	**Control (N = 9**)	**Intervention (N = 7**)	**Overall (N = 16**)
cPRA >0%, n (%)	3 (33)	0 (0)	3 (19)
HLA typing available, n (%)	7 (78)	7 (100)	14 (88)
HLA A mismatch, mean (SD)	1.3 (0.5)	1.3 (0.8)	1.3 (0.6)
HLA B mismatch, mean (SD)	1.6 (0.5)	1.6 (0.8)	1.6 (0.6)
HLA DR mismatch mean (SD)	1.3 (0.8)	0.9 (0.7)	1.1 (0.7)
Maintenance immunosuppression			
Steroids, n (%)	6 (67)	5 (71)	11 (69)
Tacrolimus, n (%)	6 (67)	4 (57)	10 (63)
Belatacept, n (%)	4 (44)	2 (29)	6 (38)
Mycophenolate mofetil or mycophenolic acid, n (%)	5 (56)	4 (57)	9 (56)
Sirolimus, n (%)	3 (33)	3 (43)	6 (38)

cPRA, calculated panel reactive antibody; SD, standard deviation.

### Feasibility and Acceptability of MyKidneyCoach

The final sample size reached was 16. This represented 40% of the target sample size of 40, all of which were recruited in 4.6 mo during the COVID-19 pandemic. Approximately 1 in 2 eligible patients agreed to participate. The withdrawal rate at the time of screening by race was 12.5% for Black patients (1/8) and 36% (5/14) for non-Black participants, including 3 of 10 White patients and 2 of 4 patients who were Asian, American Indian/Alaskan Native or did not report their race. The recruitment rate per month was 3.5 patients per month (95% CI, 2-5.6 patients per month). The overall acceptability of MyKidneyCoach by SUS score was 67.5 (95% CI, 59.1-75.9), which corresponds to grade C using the Sauro–Lewis curved grade scale.^43^ Notably, the intervention arm had a higher mean SUS score of 70.7 (95% CI, 54.8-86.6) compared with the control arm of 65 (95% CI, 53.4-76.6). Ten of 16 patients (62.5%) thought the system was easy to use. Completion rates based on the SUS, the PAM, and actively using MyKidneyCoach were 81% (13/16 patients; 95% CI, 57%-93%) for all 3 measures. Twenty-two participants completed baseline visits, and 16 completed 3-mo follow-ups with a retention rate of 73%. Patients retained in the study tended to have lower PAM scores at baseline and were older and more likely to be Black than those who withdrew from the study (**Table S1**, **SDC**, http://links.lww.com/TXD/A514).

For patients who participated in coaching (intervention arm, n = 7), on average, coaches attempted to reach participants 7 times, with participants receiving 6 coaching activities in 3 mo. The average number of meaningful coaching call activities (ie, at least 15 min) was 5.6 (SD 1) times in 3 mo. Out of the 7 participants in the intervention group, 5 of them responded to coaching activity surveys, and the average coaching helpfulness score (scale of 0–10) was 7.7 (SD 2.2). The mean total call duration in 3 mo was 215 min (SD 40 min), and the average call duration per call was 37.1 min (SD 5.3 min).

### Patient-reported Outcomes Are Improved With MyKidneyCoach and May Vary by Race

**FIGURE 2.**
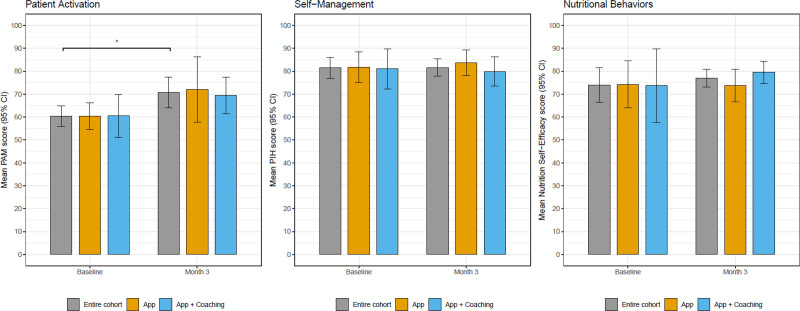
MyKidneyCoach and patient-reported outcomes (mean with SD). Shown are patient-reported outcomes of patient activation, self-management, and nutritional self-efficacy for adolescents and adult kidney transplant recipients enrolled in the pilot at baseline and 3 mo. Represented are all study participants (gray bar), attention control app only (yellow bar), and intervention app plus nurse coaching (blue bar). CI, confidence interval; PAM, patient activation measure; PIH, Partners in Health; SD, standard deviation.

**FIGURE 3.**
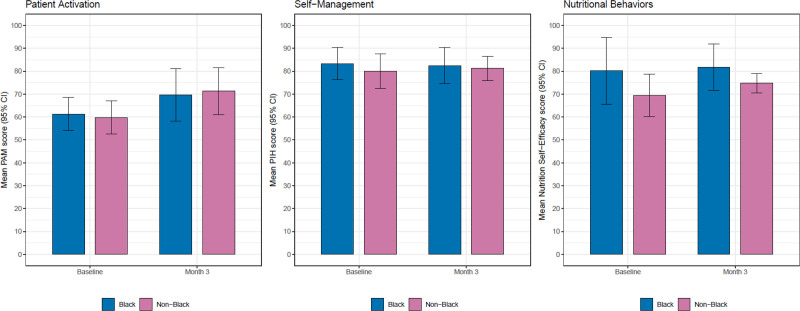
MyKidneyCoach and patient-reported outcomes by race (mean with SD). Depicted are patient activation, self-management, and nutritional self-efficacy by race (Black participants, blue bar; non-Black participants, pink bar) for the pilot study at baseline and 3 mo. CI, confidence interval; PAM, patient activation measure; PIH, Partners in Health; SD, standard deviation.

PAM significantly increased in the overall cohort for both arms, app (control), and app plus coaching (intervention) by a mean of 11 points (95% CI, 3.2-18.8) at 3 mo. The mean differences in PAM scores from baseline to 3 mo between the intervention and control arms were not significantly different (mean difference –2.4; 95% CI, –17.4 to 12.5; Figure 2A). Self-management measured by PIH did not improve from baseline to 3 mo for the entire cohort (mean difference –0.2; 95% CI, –5.5 to 5.1) and did not differ by study arms (mean group difference –3.4; 95% CI, –11 to 4.2; Figure 2B). Nutrition Self-Efficacy Scale increased by a mean of 4.4 from baseline to 3 mo for the entire cohort (95% CI, –1.7 to 10.6). The intervention arm had a 5.2 higher mean change in Nutrition Self-Efficacy Scale from baseline to 3 mo than the control arm (95% CI, –1.4 to 11.8; Figure 2C). Additionally, from the ANCOVA models, we found that Black participants tended to have a higher mean nutrition self-efficacy score of 80.5 (95% CI, 74.4-86.7) compared with 75.6 (95% CI, 71.1-80.1) in non-Black patients but lower PAM scores 69.3 (95% CI, 56.3-82.3) compared with 71.8 (95% CI, 62.5-81) in non-Black patients after 3 mo compared with baseline (Figure 3A, C). There was no difference between PIH scores for self-management between Black and non-Black patients (Figure 3B).

### Clinical Outcomes and MyKidneyCoach

The clinical outcomes by study arm are displayed in Table 3. Over the 3-mo study period, the intervention group had an improvement in hypertension compared with the control group. The 2 study groups did not differ in kidney function (measured by serum creatinine). The event rates for DSA development and allograft rejection were low. No patient experienced failed allograft or death.

**TABLE 3.** Clinical outcomes

	**Control (N = 9**)	**Intervention (N = 7**)	**Overall (N = 16**)
Hypertension (BP >130/80 mm Hg) at baseline, n (%)	5 (56)	7 (100)	12 (75)
Hypertension (BP >130/80 mm Hg) at 3 mo, n (%)	5 (56)	2 (29)	7 (44)
Missing, n (%)	0 (0)	2 (29)	2 (13)
Serum creatinine (mg/dL) at baseline, mean (SD)	1.3 (0.4)	1.3 (0.3)	1.3 (0.3)
Missing, n (%)	0 (0)	2 (29)	2 (13)
Serum creatinine (mg/dL) at 3 mo, mean (SD)	1.3 (0.3)	1.4 (0.4)	1.3 (0.3)
Missing, n (%)	0 (0)	1 (14)	1 (6)
Donor-specific antibodies at 3 mo, n (%)	1 (11.1)	0 (0)	1 (6.2)
Allograft rejection at 3 mo, n (%)	1 (11.1)	0 (0)	1 (6.2)
Allograft failure at 3 mo, n (%)	0 (0)	0 (0)	0 (0)
Patient death at 3 mo, n (%)	0 (0)	0 (0)	0 (0)

BP, blood pressure; SD, standard deviation.

### Facilitators and Barriers Regarding the Use of MyKidneyCoach

Common themes emerged, and several barriers/facilitators were identified to optimize the current intervention. Participants felt that they benefited from the coaching intervention and appreciated the coaching relationship. In particular, participants identified that through the coaching process, they were able to identify their own pathway toward change, using strategies that fit their lives, rather than receiving advice or instructions on how to successfully implement health behavior change (Table 4). One participant noted that the process of prioritizing self-identified goals and planning out incremental, progressive steps toward their goals facilitated success, stating, “*Every time she would call me, I would have this pile of things, and then I would just give them to her, I guess. She would just respond in a good way and help me how to tackle every single thing and put little goals there.*” Related to this process was the importance of the coach using a nonjudgmental communication style and facilitating change through the participant’s chosen path, as one participant noted, “*And it just made me feel that I had someone I could just explain what my situation was and not being really judged or told what to do, but more like guide me through the process of doing things by myself, for myself.*” Another participant summarized the impact of coaching as “*an amazing experience*,” and another participant noted that coaching positively impacted their life, stating, “*I honestly felt great throughout the time of the coaching. She would always call me when I needed it the most, and after I hung up, I felt I could conquer the world*.” Finally, participants shared their experiences with the mHealth app, with one participant finding that daily use of the app facilitated accountability and useful feedback to assist with tracking outcomes and monitoring their health, stating, “*I love that app. It kept me on my toes. It was something that was a constant reminder: did you take your medicine? How are you feeling? If my stress level was high or I was fatigued, it was me getting it out of my head and putting it in an app*.”

**TABLE 4.** Barriers and facilitators to use of MyKidneyCoach intervention

**Theme**	**Quotation**
Facilitators of use and success of intervention	
Self-management skill development	*Because even though I come from a huge family, it’s very different when you don’t know this person that much and this person gives you all the tools, and it’s not really telling you because they love you or because they have to support you, but they’re telling you because … I don’t know. It’s just life skills.*
Prioritization and planning small behavioral steps	*Every time she would call me, I would have this pile of things, and then I would just give them to her, I guess. She would just respond in a good way, and help me how to tackle every single thing and put little goals there.*
Nonjudgmental environment and self-identified goals and solutions	*I just felt that she had so much experience and that she understood what I was saying and going through. And it just made me feel that I had someone I could just explain what my situation was and not being really judged or told what to do, but more like guide me through the process of doing things by myself, for myself.*
Tracking outcomes and behaviors through app	*I love that app. It kept me on my toes. It was something that was a constant reminder: did you take your medicine? How are you feeling? If my stress level was high or I was fatigued, it was me getting it out of my head and putting it in an app.*
Barriers to use of app and coaching intervention	
Lack of communication between app and healthcare team	*The only thing I would have tweaked in the app is that for a couple of days I saw that there was a lot of fatigue, a lot of stress. I think it’s something that should go to a doctor or an advisor that can give you a little advice to say, ‘okay. We need you to relax. Just relax. Have you taken your medicine?*
App—not user-friendly	*I love the concept, [but] it didn’t talk to other apps. It was pretty clunky, so I gave up after 2 of 3 times of trying to use it. … It wasn’t user-friendly or intuitive.*
Insufficient duration	*I felt like we could have spent more time…. Maybe if we had a longer period, it would be nice.*

Participants also noted barriers to the use of the app. One participant suggested that the app would be more useful if it could communicate with their electronic health record and alert providers to concerning patterns, sharing, “*for a couple of days I saw that there was a lot of fatigue, a lot of stress. I think it’s something that should go to a doctor or an advisor that can give you a little advice.*” Another participant characterized the app as difficult to navigate and therefore stopped using the app, stating, “*I love the concept, [but] it didn’t talk to other apps. It was pretty clunky, so I gave up after 2 or 3 times of trying to use it. … It wasn’t user-friendly or intuitive*.” When asked about the coaching intervention, the only improvement noted was to lengthen the duration of coaching to continue the receipt of social support and explore more health behavior goals.

## DISCUSSION

We found that the use of MyKidneyCoach was acceptable in diverse KT patients. Recruitment, especially achieving the target enrollment, was impacted by the COVID-19 pandemic. Interestingly, Black patients had a lower withdrawal rate at the time of screening than non-Black patients, which needs to be further explored. Despite our recruitment challenges, we gained important initial insights from this pilot for future studies. Importantly, the coaching aspect of the intervention arm was well accepted, with improved PAM scores, nutrition self-efficacy scores, and hypertension compared with the attention control (app alone). Furthermore, Black transplant recipients were more likely to have higher nutrition self-efficacy, despite being less activated compared with their White counterparts, signifying that MyKidneyCoach benefits may differ by race.^3-10,12,13^ Taken together, these data support the potential role of MyKidneyCoach to improve patient activation and clinical outcomes in diverse populations. Understanding the contextual determinants of discordant recruitment, patient activation, and self-efficacy demonstrated in Black participants for future iterations of MyKidneyCoach is essential to mitigate racial disparities in KT.

The clinical consequences of inadequate patient activation and self-management in KT are extensive and disproportionately affect Black recipients. Even after accounting for potential immunological differences, Black recipients have higher rates of rejection episodes and allograft failure compared with White recipients. A variety of social and environmental factors leading to poor patient activation and self-management have been shown to be major contributing factors.^5,14^ Black recipients have been found more likely to live in low-income households and face challenges in posttransplant self-care, such as taking immunosuppressant medication, than non-Black recipients.^44-48^ Social determinants of health, such as low health literacy, prioritization of other needs over medications, and racism in the transplant process, have been implicated.^49-51^ Importantly, one study demonstrated that low medication self-management in Black recipients was attenuated when stratified by transplant center.^52^ This demonstrates the potential role of the local transplant team to work within contextual factors that influence posttransplant self-management, provide resources to alleviate inequities, and improve clinical outcomes in KT.^52^

In our pilot study, both Black and White participants benefited from the use of MyKidneyCoach, with an increase in their patient activation and a reduction in hypertension. Notably, Black participants had an improvement in their nutrition self-efficacy compared with their White counterparts, which may be a reflection of minoritized populations preferentially using and responding to mHealth modalities^24^ for their medical care. This is consistent with previous studies that have shown either mHealth or coaching can play a critical role in improving patient activation and self-management in Black patients with chronic disease.^21,22,53^ In-person, health educator coaching reduced racial inequities in pain control for Black cancer patients, whereas an mHealth medication safety intervention improved blood pressure control in Black KT recipients (19–20). Additionally, short-term studies in heart transplantation and KT demonstrated that mHealth led to improvements in medication adherence and hypertension management across racial groups (32–34). Our use of MyKidneyCoach is the first study to combine mHealth with tailored coaching through an app, which capitalizes on minority participants being the most active users of the mobile web.^24^

Prior interventions in solid organ transplantation have predominantly focused on only certain aspects of posttransplant self-monitoring, such as medication adherence, hypertension, or blood sugar monitoring. These interventions used in-person coaching, educational content, or text-based medication reminders.^22,54-69^ This resulted in modest improvement in medication adherence but significant attrition because of notification burn-out and lack of personalized 2-way communication. Most interventions were solely focused on one aspect of self-management dictated by the study team, which may not have coincided with the patient’s own self-management goals. Unlike previous studies, participants in our study had a completion rate of 81% for app usage and an adequate retention rate of 73%, especially for Black transplant recipients, further supporting the use of mHealth technologies, such as MyKidneyCoach, in minoritized populations.^24^ Those in the intervention group with app plus coaching had higher SUS scores and actively participated in the coaching. In our study, the tailored coaching helped participants engage in their care and use the app more as they chose self-determined goals rather than being dictated by the medical team in a paternalistic way.

Although our pilot showed promising and beneficial results, there were some limitations. First, our sample size was small and recruitment was affected by the COVID-19 pandemic. Because of a lack of power, we were unable to determine which modality app alone or app plus coaching would be more beneficial to reduce racial disparities. Additionally, we could not analyze the optimum time posttransplant to use the app. Second, the stratified randomization of the age group did not result in a balance in age distribution between the 2 groups, as few patients recruited were <21 y old. Third, feedback from participants showed that the mHealth monitoring app would be more user-friendly if it allowed participants to focus on specific self-determined posttransplant management skills by turning on or off functionality, instead of having access to all aspects of self-monitoring, which appeared overwhelming. Finally, we found that although Black participants increased their patient activation with MyKidneyCoach, this was less than their White counterparts. An important factor that may have contributed to this difference is the type of coach. Some studies have shown that Black patients respond better to racially concordant providers,^70^ whereas other studies revealed that peer coaching may be beneficial.^71,72^ Unfortunately, we were unable to explore these aspects of discordance between patient activation and self-efficacy because of the pilot nature of the study. The contextual mechanisms that contribute to this discordance are critically important in addressing transplant racial inequities. Future studies are essential to determine the efficacy of a mHealth app alone or paired with coaching, the most effective coaching modality, the effect of racial concordance on coaching, and optimal timing for app use to improve equity for Black transplant recipients.

In conclusion, MyKidneyCoach, involving self-monitoring app with or without coaching, was easy to use, the coaching component was readily accepted, and improvements were demonstrated in patient-reported outcomes. Both Black and non-Black participants benefited from the intervention. This highlights the potential use of our tailored mHealth intervention and identifies aspects requiring further investigation to make MyKidneyCoach a more effective intervention to reduce healthcare inequities in KT.

## ACKNOWLEDGMENTS

The authors extend special thanks to all study participants and their families.

## Supplementary Material
