# Supplementary material for: MyKidneyCoach, Patient Activation, and Clinical Outcomes in Diverse Kidney Transplant Recipients: A Randomized Control Pilot Trial
Source: Transplant Direct. 2023 Mar 15;9(4):e1462. doi: 10.1097/TXD.0000000000001462 (PMC10019211; doi:10.1097/TXD.0000000000001462)
Supplement: Supplementary file 1 [file txd-9-e1462-s001.pdf]

Table S1. Patient characteristics by study withdrawal			
	Withdrew after randomization (N= 6)	Remained in the study (N= 16)	Overall (N= 22)
**PAM Score**			
**Mean (SD)**	63 (7.7)	60 (8.5)	61 (8.2)
**Median [Q1, Q3]**	65 [63, 68]	63 [55, 68]	63 [56, 68]
**[Min, Max]**	[49, 70]	[41, 70]	[41, 70]
**PAM Level**			
**Mean (SD)**	2.8 (0.41)	2.7 (0.60)	2.7 (0.55)
**Median [Q1, Q3]**	3.0 [3.0, 3.0]	3.0 [2.8, 3.0]	3.0 [3.0, 3.0]
**[Min, Max]**	[2.0, 3.0]	[1.0, 3.0]	[1.0, 3.0]
**Age at Consent**			
**Mean (SD)**	27 (13)	38 (12)	35 (13)
**Median [Q1, Q3]**	27 [16, 38]	43 [35, 48]	41 [23, 45]
**[Min, Max]**	[14, 43]	[14, 50]	[14, 50]
**Sex**			
**Male**	4 (67%)	8 (50%)	12 (55%)
**Female**	2 (33%)	8 (50%)	10 (45%)
**Race**			
**Black**	1 (17%)	7 (44%)	8 (36%)
**Other**	2 (33%)	2 (13%)	4 (18%)
**White**	3 (50%)	7 (44%)	10 (45%)
**Ethnicity**			
**Non-Hispanic**	6 (100%)	15 (94%)	21 (95%)
**Hispanic or Latino**	0 (0%)	1 (6%)	1 (5%)
**Unknown**	0 (0%)	0 (0%)	0 (0%)
